# Trends of under-five mortality and associated risk factors in Zambia: a multi survey analysis between 2007 and 2018

**DOI:** 10.1186/s12887-022-03362-7

**Published:** 2022-06-13

**Authors:** Amanuel Kidane Andegiorgish, Henok G. Woldu, Mohamed Elhoumed, Zhonghai Zhu, Lingxia Zeng

**Affiliations:** 1 Department of Epidemiology & Biostatistics, School of Public Health, Xi’an Jiaotong University Health Science Center, 76 West Yanta Road, Xi’an, 710061 Shaanxi Province China; 2 School of Public Health, Asmara College of Health Sciences, Asmara, Eritrea; 3 Department of Epidemiology and Biostatistics, College of Public Health, University of Georgia, Athens, GA USA; 4 National Institute of Public Health Research (INRSP), BP. 695, Nouakchott, Mauritania; 5 Key Laboratory of Environment and Genes Related to Diseases Xi’an Jiaotong University, Ministry of Education, Xi’an, Shaanxi 710061 People’s Republic of China

**Keywords:** Children, Mortality, Determinants, Demographic and Health Survey, Zambia

## Abstract

**Background:**

Mortality at a young age is key to public health measures. This study aims to describe the burden, trend, and associated factors of under-five mortality rate (U5MR) in Zambia from 2007-to-2018.

**Method:**

A sample of 29,274 children under-five were analyzed from the Zambia demographic and health survey (ZDHS). Univariate and bivariate analysis were used to identify factors influencing U5M.

**Result:**

Pooled prevalence of U5MR in Zambia was 84.4/ 1000 live-births. Over 15 years, U5M has declined by 49% (from 118.7 to 60.5/1000 live-births). Compared to children of teenage (≤19 years) mothers the likelihood of U5M was lower by 24 to 37% among children of 20 to 34 years old mothers. The likelihood of U5M was lower by 23% (AOR, 0.77 95%CI, 0.58–1.04) for poorest, 27% (AOR, 0.73 95%CI, 0.55–0.98) for poorer, and 19% (AOR, 0.81 95%CI, 0.62–1.07) for middle as compared to the richest households. The likelihood of U5M was 21% (AOR, 0.79 95%CI, 0.67–0.93) lower among rural residents. Multiple-born children died 2.54 times (95%CI, 1.95–3.98) higher than the single-born. Male children (AOR, 1.28, 95% CI, 1.23–1.46), smaller than average birth size (AOR, 1.78; 95% CI, 1.52–2.09), and no ANC visit (AOR, 3.17, 95% CI, 2.74–3.67) were associated with U5M. The likelihoods of U5M were significantly higher in the Eastern, Luapula, and Muchinga regions than in the Central.

**Conclusion:**

This study revealed that Zambia has made a gain on child survival. Further efforts targeting mothers, children, and provinces are needed to scale up the decline and achieve the SDG3.

**Supplementary Information:**

The online version contains supplementary material available at 10.1186/s12887-022-03362-7.

## Introduction

Over the past several decades the world has recorded remarkable progress on child survival reflecting regions, countries, and communities’ commitment towards the global agenda of improving children’s life [1–4]. Under-five mortality (U5M) has dropped by almost 60% since 1990 globally despite the 5.2 million children died in 2019 alone before reaching their fifth birthday primarily due to infectious diseases, which can be avoided by practicing healthy behaviors. This global decline in U5M from 93/1000 live births in 1990 to 38/1000 live births in 2019 has spared more than 20,000 lives per day (14,000 deaths/day in 2019 vs 34,000 deaths/day in 1990) [1].

Despite all evidence illustrating how substantial investments have been implemented to avert associated U5M and the global success towards improving the lives of children, they continue to face widespread disparities in their chance of survival [4, 5]. Sub-Saharan Africa (SSA) remains the region with the highest U5M in the world, 76 deaths/1000 live births, which is equivalent to 1 child in 13 dying before reaching the age of 5. This rate is 20 times higher than that of 1 in 264 in the region of Australia and New Zealand and more than two decades behind the world average, which was achieved at a rate of 1 in 13 by 1999 [1, 3]. Limited access to public health interventions is a major factor for poor child health and survival outcomes in developing countries [6]. Therefore, information on disparities in child survival at the country level should form the basis of targeted interventions in the poorest subpopulations [7].

Over the years up to 2016, the eastern and southern part of the SSA region had greater improvement in U5M [7]. Zambia is a land-locked SSA country with more than 17.9 million population in the southern part of Africa. In 2016 Zambia was home to more than 11.6% of its people living with Human Immunodeficiency Virus (HIV) [8]. Malaria and respiratory infections were attributable to 81% of U5M [9]. Although substantial improvement in U5M was achieved from 1990 to 2019, this reduction was lower than some SSA countries [10] and Zambia remains one of the 73 countries pacing below the sustainable development goal (SDG) targets on U5M [11].

Under-five mortality is a key indicator of the extent of survival and economic development on interventions towards the overall well-being of a country. Therefore, proper comprehension of U5M requires a deeper understanding of the tolls on the prevailing levels and trends of mortality by years, regions as well as countries [5, 12]. Overall U5M is reported in comparison with the previous surveys in the Demographic and health survey (DHS), and this can hinder on the identification of the modifiable factors which might differ on each round and may mask the priority targets for intervention. Therefore, an estimated report of the segregated and pooled data in this study will provide better information that public health researchers and policymakers could use for evidence-based interventions.

Although, several studies provided useful insights into the determinants of U5M, which differ in their effects across sociodemographic and regions [4, 5, 13–15]. DHS data represents a more reliable source for identifying the risk factors of U5M [15]. The representative nature of DHS data is useful for designing prevention programs. Assessing time trend mortality by cause is essential in guiding the lasting effect of appropriate child health intervention programs [16]. Therefore, this study aimed to describe trends of under-five mortality (U5M) and associated factors over a decade (2007–2018) in Zambia.

## Methods and materials

### Study design

This study was a population-based retrospective, cross-sectional design using secondary data of three Zambia demographic and health surveys; ZDHS 2007, ZDHS 2013–14, and ZDHS 2018.

### Data sources

We retrieved the children component data from the latest three consecutive Zambia demographic and health surveys (ZDHSs) (2007, 2013–14, and 2018) to investigate the trends and associated factors of U5M. DHS is a national representative five-year periodic survey used to collect information from women aged 15–49 years and men aged 15–59 years about demographic and health status. The DHS uses an international standard tool for data collection using a stratified two-stage cluster designs in which urban and rural place of residence were considered as strata. Then census enumeration areas were used as clusters among urban and rural areas. Later a complete listing of households was served as a sampling frame for selecting households to be interviewed. Information on the key components of birth histories and background characteristics of all live births 5 years preceding the date of interview were collected from the women. Details of the DHS are published elsewhere [17].

### Outcome variables

The primary outcome of our study was U5M (dichotomized as yes or no) during the 5 years preceding the survey. To explore the association of independent variables on U5M, the assumption of the unequal contribution of Children’s birth cohorts at different times to the denominator of U5M calculation was fully considered. All newborns do not contribute equally to the U5M calculation to the DHS surveys. Therefore, the pooled prevalence estimate of U5MR among the three surveys of Zambia was calculated using the DHS.rates R package in R software for each survey separately. The DHS.rates in R packages calculates the point estimates of all neonatal, infant, child, and under-five mortalities with their standard error [18]. After calculating the U5MR with their standard error for each survey, data were extracted in excel and imported to STATA 17, then pooled prevalence of U5MR in Zambia and the regions were estimated using the “metan” STATA command.

### Explanatory variables

To identify the potential factors influencing U5M in Zambia, we have adopted the Mosley and Chen framework of factors influencing child survival in developing countries [19], which was the basis for selecting potential risk factors for childhood mortality. A set of sociodemographic variables related to the U5M were identified from the survey data sets classified as maternal, child, or regional factors. Maternal age in years, marital status, education, occupation, household wealth index, place of residence, antennal care (ANC) visit and timing of antennal care visit during the index pregnancy, place of delivery, birth type, birth size, sex, and order (parity), as well as region were used as predictors of U5M.

### Statistical analysis

Data were cleaned and analyzed using STATA/MP,V.17.0 (StataCorp). Continuous variables like age and years of education attended were grouped into categories. All analysis was conducted using weight, clustering, and stratification variables provided by the DHS (sampling weight (V005), primary sampling unit (V023), and strata (V021)), using the “svy” command, to account for adjustment by strata and study design. Descriptive analysis was used to summarize the background characteristics. Chi-square tests were used to analyze the potential factors associated with U5M.

A multilevel regression model was fitted to account for the clustered data structure using a mixed effect logistic regression (fixed and random effects) using cluster (region) as a random variable. Likelihood Ratio test (LR) and Intra-class Correlation Coefficient (ICC) were estimated for four fitted models including the null model (a model without explanatory variables), and the year of the survey was treated as fixed effects “i.survey” in the model specification. The ICC [20] (a measure of the heterogeneity of U5MR among clusters attributed to cluster variation) of the clustered data was found very negligible, and one level ordinary logistic regression was employed to investigate the association between independent variables and the outcome. Then, we fitted a multivariable logistic regression model that included all variables with complete case analysis and a *P-*value < 0.20 in the bivariate analysis. A two-sided alpha level of 0.05 was used to determine statistical significance in all the analyses.

## Result

### Characteristics of study participants

This analysis included 29,274 children under-5 years old over the latest three DHS surveys (6467 from 2007, 13,251 from 2013 to 14 and 9556 from 2018) (Table 1).

**Table 1:** Background characteristics of study participants and U5M by year of survey (weighted)

	Survey-2007		Survey-2013_14		Survey-2018	
Variables	***N*** = 6467	U5M (%)	***N*** = 13,251	U5M (%)	***N*** = 9556	U5M (%)
**Maternal Factors**						
**Maternal age**						
15–19	416 (6.43)	40 (9.62)	962 (7.26)	59 (6.13)	826 (8.64)	43 (5.21)
20–24	1673 (25.87)	169 (10.10)	3181 (24.01)	190 (5.97)	2545 (26.63)	145 (5.70)
25–29	1846 (28.54)	177 (9.59)	3447 (26.01)	165 (4.79)	2237 (23.41)	92 (4.11)
30–34	1308 (20.23)	109 (8.33)	2745 (20.72)	153 (5.57)	1856 (19.42)	81 (4.36)
35–39	757 (11.71)	74 (9.78)	1823 (13.76)	99 (5.43)	1276 (13.35)	53 (4.15)
40–49	467 (7.22)	54 (11.56)	1093 (8.25)	75 (6.86)	816 (8.54)	43 (5.27)
**Marital status**						
Married	6014 (93.0)	577 (9.59)	12,109 (91.38)	686 (5.67)	8436 (88.28)	401 (4.75)
Not married	453 (7.0)	46 (10.15)	1142 (8.62)	55 (4.82)	1120 (11.72)	56 (5.00)
**Maternal education**						
No education	856 (13.24)	84 (9.81	1487 (11.23)	96 (6.46)	1029 (1.77)	54 (5.25)
Primary	4042 (62.50)	397 (9.82)	7370 (55.66)	405 (5.50)	4976 (52.07)	232 (4.66)
Secondary & higher	1569 (24.26)	142 (9.05)	4383 (33.10)	239 (5.45)	3551 (37.16)	171 (4.82)
**Maternal occupation**						
Not working	2611 (40.45)	247 (9.46)	5071 (38.88)	294 (5.80)	4246 (44.45)	211 (4.97)
Working	3844 (59.55)	375 (9.76)	7971 (61.12)	435 (5.46)	5306 (55.55)	246 (4.64)
**Wealth index**						
Poorest	1401 (21.66)	125 (8.92)	3167 (23.90)	183 (5.78)	2732 (28.59)	142 (5.20)
Poorer	1406 (21.74)	131 (9.32)	3183 (24.02)	170 (5.34)	2309 (24.16)	102 (4.42)
Middle	1481 (22.90)	139 (9.39)	3004 (22.67)	179 (5.96)	1879 (19.66)	90 (4.79)
Richer	1365 (21.11)	145 (10.62)	2242 (16.92)	133 (5.93)	1408 (14.73)	64 (4.55)
Richest	814 (12.59)	83 (10.63)	1655 (12.49)	76 (4.59)	1228 (12.85)	59 (4.80)
**Place of residence**						
Urban	2096 (32.41)	223 (10.64)	4897 (36.96)	293 (5.98)	2822 (29.53)	139 (4.93)
Rural	4371 (67.59)	400 (9..15)	8354 (63.04)	448 (5.36)	6734 (70.47)	318 (4.72)
**ANC**						
No ANC visits	2458 (38.01)	378 (15.38)	4196 (31.67)	454 (10.82)	2486 (26.02)	243 (9.77)
Had at least one ANC	4009 (61.99)	245 (6.11)	9055 (68.33)	287 (3.17)	7070 (73.98)	214 (3.03)
**Timing of the first ANC visit**						
Less than 12 weeks	754 (11.66)	39 (5.17)	2186 (16.50)	80 (3.66)	2712 (28.38)	94 (3.47)
Greater than 12 weeks	5713 (88.34)	584 (10.22)	11,065 (83.50)	661 (5.97)	6844 (71.62)	363 (5.30)
**Place of delivery**						
Institutional delivery	3145 (49.13)	282 (8.97)	9046 (68.56)	464 (5.13)	7890 (82.57)	360 (4.56)
Home delivery	3256 (50.87)	275 (8.45)	4148 (31.44)	246 (5.93)	1666 (17.43)	97 (5.82)
**Child characteristics**						
**Birth type**						
Singleton	6193 (95.76)	552 (8.91)	12,833 (96.85)	656 (5.11)	9252 (96.82)	411 (4.44)
Multiple birth	274 (4.24)	71 (25.91)	418 (3.15)	85 (2.33)	304 (3.18)	46 (15.13)
**Size of child at birth**						
Average	3551 (56.17)	282 (7.94)	7723 (59.43)	369 (4.78)	5833 (62.75)	87 (7.71)
Smaller than average	721 (11.40)	97 (13.45)	1506 (11.59)	138 (9.16)	1129 (12.15)	248 (4.25)
Larger than average	2050 (32.43)	155 (7.56)	3766 (28.98)	177 (4.70)	2333 (25.10)	97 (4.16)
**Child sex**						
Male	3216 (49.73)	345 (10.73)	6721 (50.72)	402 (5.98)	4752 (49.73)	249 (5.24)
Female	3251 (50.27)	278 (8.55)	6530 (49.28)	339 (5.19)	4804 (50.27)	208 (4.33)
**Birth order**						
First	1301 (20.12)	146 (11.22)	2824 (21.31)	190 (6.73)	2399 (25.10)	132 (5.50)
Second−third	2267 (35.05)	220 (9.70)	4361 (32.91)	226 (5.18)	3243 (33.94)	153 (4.72)
Fourth & above	2899 (44.83)	257 (8.87)	6066 (45.78)	325 (5.36)	3914 (40.96)	172 (4.39)
**Regions**						
Central	616 (9.53)	53 (8.60)	1147 (8.66)	52 (4.53)	956 (10.00)	39 (4.08)
Copperbelt	634 (9.80)	55 (8.68)	1140 (8.60)	68 (5.96)	874 (9.15)	31 (3.55)
Eastern	909 (14.06)	101 (11.11)	1613 (12.17)	123 (7.63)	1153 (12.07)	52 (4.51)
Luapula	723 (11.18)	71 (9.82)	1545 (11.66)	99 (6.41)	1173 (12.28)	85 (7.25)
Lusaka	651 (10.07)	69 (10.60)	1163 (8.78)	58 (4.99)	1008 (10.55)	48 (4.76)
Northern	796 (12.31)	99 (12.44)	1514 (11.43)	93 (6.14)	950 (9.94)	48 (5.05)
North-western	718 (11.10	53 (7.38)	1362 (10.28)	65 (4.77)	770 (8.06)	19 (2.47)
Southern	746 (11.54)	65 (8.71)	1470 (11.09)	76 (5.17)	990 (10.36)	38 (3.84)
Western	674 (10.42)	57 (8.46)	1063 (8.02)	45 (4.23)	809 (8.47)	40 (4.94)
Muchinga	–	–	1234 (9.31)	62 (5.02)	873 (9.14)	57 (6.53)

*U5M* under 5 years’ mortality

Age distribution of mothers was consistent across the three ZDHSs. More unmarried mothers were interviewed in the 2018 survey (11.7%) than in 2013–14 survey (8.6%) and the 2007 surveys (7.0%).

Majority of the participants had a primary level of education (> 50%). Attaining secondary and above educational level has increased from 24.3% in 2007 to 37.2% in 2018, while the percentage of non schooling at all has declined from 13.2 to 1.8% over the same time period. More than half (55.6%) of all women in the three surveys had work outside home. Distribution of mothers across the five categories of wealth index was similar with more than 43% of the respondents below the middle class. More than two-thirds of the mothers were from a rural place of residence. Majority (> 60%) of the women had at least one ANC visit during the index pregnancy. Practice of institutional delivery has increased from less than half (49.1%) in the 2007 survey to 82.6% in the 2018 survey. The proportion of multiple births among the interviewed women was very small (< 5%) and more than 56% of all newborns were on average birth size at birth. Nearly half (40%) of all the children in all the surveys were born of fourth and above birth order (Table 1).

Ten regions were stratified in the latest two (2013–14 and 2018) surveys, while nine in the 2007 survey. Participants’ proportion was comparable in all the regions across the three surveys (Table 1).

### Factors associated with U5M in Zambia

Table 2 shows the pooled data distribution of the variables under study for U5M and its association. Tests have shown a statistically significant association between U5Ms and survey years, maternal education, ANC visit, place of delivery, the plurality of birth (birth type), birth size, sex of the child, birth order, and some regions of Zambia (Table 2).

**Table 2:** Association of independent variables with under five mortality (weighted) in Zambia

Variables	N (%) 29,274	Pooled U5M		X^**2**^ -value	***P***-value
		Alive (%)	Dead (%)		
		27,453	1821		
**Survey year**				56.03	< 0.001
2007	6467 (22.4)	5844 (90.0)	623 (10.0)		
2013–14	13,251 (45.3)	12,510 (94.3)	741 (5.7)		
2018	9556 (32.4)	9099 (95.1)	457 (4.9)		
**Maternal Factors**					
**Maternal age**				1.71	0.132
15–19	2204 (7.4)	2062 (93.6)	142 (6.4)		
20–24	7399 (25.2)	6895 (93.2)	504 (6.8)		
25–29	7530 (25.7)	7096 (94.2)	434 (5.8)		
30–34	5909 (20.1)	5566 (94.2)	343 (5.8)		
35–39	3856 (13.6)	3630 (94.1)	226 (5.9)		
40–49	2376 (8.0)	2204 (92.7)	172 (7.3)		
**Marital status**				0.24	0.622
Not married	2715 (8.4)	2558 (94.2)	157 (5.8)		
Married	26,559 (91.6)	24,895 (93.7)	1664 (6.3)		
**Maternal education**				3.09	0.047
No education	3372 (11.4)	3138 (93.1)	234 (6.9)		
Primary	16,388 (56.1)	15,354 (93.7)	1034 (6.3)		
Secondary & higher	9503 (32.5)	8951 (94.2)	552 (5.8)		
**Maternal occupation**				0.25	0.612
Not working	11,928 (42.4)	11,176 (93.7)	752 (6.3)		
Working	17,121 (57.6)	16,065 (93.8)	1056 (6.2)		
**Wealth index**				1.35	0.251
Poorest	7300 (24.5)	6850 (93.8)	450 (6.2)		
Poorer	6898 (22.6)	6495 (94.2)	403 (5.8)		
Middle	6364 (20.0)	5956 (93.6)	408 (6.4)		
Richer	5015 (18.2)	4673 (93.2)	342 (6.8)		
Richest	3697 (14.7)	3479 (94.1)	218 (5.9)		
**Place of residence**				0.68	0.409
Urban	9815 (33.2)	9160 (93.3)	655 (6.7)		
Rural	19,459 (66.8)	18,293 (94.0)	1166 (6.0)		
**ANC**				576.9	< 0.001
No ANC visits	9140 (31.1)	8065 (88.2)	1075 (11.8)		
Had at least one ANC	20,134 (68.9)	1388 (96.3)	746 (3.7)		
**Timing of the first ANC visit**				61.8	< 0.001
Less than 12 weeks	5662 (19.31)	5439 (96.23)	213 (3.77)		
Greater than 12 weeks	23,622 (80.69)	22,014 (93.19)	1608 (6.81)		
**Place of delivery**				10.7	0.001
Institutional delivery	20,081 (68.6)	18,975 (94.5)	1106 (5.5)		
Home delivery	9070 (31.4)	8452 (93.2)	618 (6.8)		
**Child characteristics**					
**Birth type**				187.1	< 0.001
Singleton	28,278 (96.7)	26,659 (94.3)	1619 (5.7)		
Multiple birth	996 (3.3)	794 (79.7)	202 (20.3)		
**Size of child at birth**				38	< 0.001
Average	17,107 (59.79)	16,208 (94.74)	899 (5.26))		
Smaller than average	3356 (11.73)	3034 (90.41	322 (9.59)		
Larger than average	8149 (28.48)	7720 (94.74)	429 (5.26)		
**Child sex**				14.5	< 0.001
Male	14,689 (50.4)	13,693 (93.2)	996 (6.9)		
Female	14,585 (49.6)	13,760 (94.3)	825 (5.7)		
**Birth order**				4.9	0.007
First	6524 (22.3)	6056 (92.8)	468 (7.2)		
Second−fourth	9871 (34.0)	9272)(93.9)	599 (6.1)		
Fifth & above	12,879 (43.7)	12,125 (94.1	754 (5.9)		
**Regions**				4.0	< 0.001
Central	2719 (9.3)	2575 (94.7)	144 (5.3)		
Copperbelt	2648 (9.1)	2494 (94.2)	154 (5.8)		
Eastern	3675 (12.6)	3399 (92.5)	276 (7.5)		
Luapula	3441 (11.8)	3186 (92.6)	255 (7.4)		
Lusaka	2822 (9.6)	2647 (93.8)	175 (6.2)		
Northern	3182 (10.9)	2988 (93.9)	194 (9.1)		
North-western	2878 (9.8)	2729 (94.8)	149 (5.2)		
Southern	3134 (10.7)	2963 (94.5)	171 (5.5)		
Western	1872 (6.4)	1787 (95.5)	85 (4.5)		
Muchinga	2903 (9.9)	2685 (92.5)	218 (7.5)		

Muchinga was part of the Northern region in the 2007 survey

### Univariate analysis of factors associated with U5M in Zambia

Univariate logistic regression indicated that the likelihood of U5M was lower among children born from mothers 20–34 years old across the three surveys. Children of unmarried mothers had experienced lower U5M except in the 2018 survey where U5M was 1.24 times higher among children of unmarried women. Pooled data indicated that the likelihood of U5M was 1.22 time and 1.16 times higher among non-schooled and only primary level of education attended mothers compared to secondary and above and the difference was significant. Children of working mothers had a 10% higher likelihood of U5M than non-working in the 2007 survey. The likelihood of U5M was higher among the lower household wealth status group in the 2013–14 survey. This was the opposite in the 2007 and 2018 surveys in which children of lower household wealth group had 4–8% lower likelihood of dying than the richest group. U5M was 5% lower among children from rural areas than from urban areas. Antenatal care visits and timing (first trimester) ANC attendance had a significant association with U5M in Zambia across the three ZDHS surveys.

U5M was more than 4.2 times higher among multiple births than single births, and the difference was statistically significant. Children born lower than average birth sizes had a significantly higher likelihood of dying before celebrating their fifth birthday than the average birth size. Similarly, male children had significantly higher U5M compared to their female counterparts. Furthermore, the likelihood of U5M was 1.25 times higher among children of first-time birth mothers (primigravida) compared to fourth and above birth orders.

Compared to the Central province, the likelihood of U5M was higher in almost all the other provinces of Zambia. The difference was significant in Eastern, Luapula, and Muchinga provinces.

### Multivariate analysis of factors associated with U5M in Zambia

Table 3 indicates that compared to the 2007 ZDHS, U5M was 33% lower (AOR, 0.67, 95% CI, 0.58–10.78) and 37% lower (OR, 0.63, 95% CI, 0.53–0.76) in the 2013–14 and 2018 surveys respectively.

**Table 3:** Multivariable analysis of U5M and associated factors in Zambia, using the recent three ZDHS surveys

Variables	ZDHS-2007	ZDHS-2013-14	ZDHS-2018	Pooled ZDHS-2007 to 2018
	AORs(95%CI)	AORs(95%CI)	AORs(95%CI)	AORs(95%CI)
**Maternal Factors**				
**Maternal age**				
15–19	**Reference**	**Reference**	**Reference**	**Reference**
20–24	0.71 (0.46,1.09)	0.63 (0.41,0.96)*	1.00 (0.59,1.69)	0.74 (0.56,0.96)*
25–29	0.66 (0.42,1.05)	0.61 (0.37,1.02)	0.60 (0.29,1.23)	0.61 (0.44,0.84)**
30–34	0.65 (0.35,1.22)	0.71 (0.40,1.24)	0.60 (0.26,1.36)	0.63 (0.43,0.92)*
35–39	0.95 (0.47,1.91)	0.86 (0.46,1.64)	0.68 (0.25,1.88)	0.79 (0.51,1.23)
40–49	1.16 (0.57,2.35)	1.01 (0.53,1.92)	1.05 (0.41,2.70)	0.99 (0.64,1.54)
**Marital status**				
Married	**Reference**	**Reference**	**Reference**	**Reference**
Not married	⁋	⁋	1.48 (0.91,2.41)	⁋
**Maternal education**				
No education	0.96 (0.63,1.45)	1.05 (0.73,1.50)	0.92 (0.58,1.48)	0.99 (0.78,1.25)
Primary	1.12 (0.85,1.47)	0.94 (0.71,1.24)	1.01 (0.65,1.52)	0.99 (0.82,1.20)
Secondary & higher	**Reference**	**Reference**	**Reference**	**Reference**
**Maternal occupation**				
Not working	**Reference**	**Reference**	**Reference**	**Reference**
Working	⁋	1.01 (0.81,1.25)	⁋	⁋
**Wealth index**				
Poorest	0.76 (0.45,1.28)	1.25 (0.73,2.16)	0.60 (0.32,1.11)	0.77 (0.58,1.04)
Poorer	0.86 (0.49,1.48)	1.15 (0.68,1.96)	0.53 (0.30,0.94)	0.73 (0.55,0.98)*
Middle	0.91 (0.56,1.45)	1.17 (0.73,1.87)	0.68 (0.38,1.22)	0.81 (0.62,1.07)
Richer	1.17 (0.85,1.62)	1.42 (0.89,2.27)	1.06 (0.65,1.73)	1.17 (0.91,1.49)
Richest	**Reference**	**Reference**	**Reference**	**Reference**
**Place of residence**				
Urban	**Reference**	**Reference**	**Reference**	**Reference**
Rural	0.83 (0.58,1.20)	0.81 (062,1.05)	0.88 (0.59,1.31)	0.79 (0.67,0.93)**
**ANC visits**				
Had at least one ANC visits	**Reference**	**Reference**	**Reference**	**Reference**
No ANC	2.33 (1.94,2.81)***	3.50 (2.80,4.38)***	4.13 (2.92,5.83)***	3.17 (2.74,3.67)***
**Timing of the first ANC visit**				
< 12 weeks	**Reference**	**Reference**	**Reference**	**Reference**
> 12 weeks	1.14 (0.80,1.61)	0.94 (0.70,1.27)	0.81 (0.59,1.12)	0.96 (0.80,1.15)
**Place of delivery**				
Institutional delivery	**Reference**	**Reference**	**Reference**	**Reference**
Home delivery	1.00 (0.77,1.30)	1.13 (0.90,1.41)	1.08 (0.78,1.49)	1.08 (0.92,1.24)
**Child Characteristics**				
**Birth type**				
Singleton	**Reference**	**Reference**	**Reference**	**Reference**
Multiple birth	2.65 (1.73,4.06)***	2.66 (1.78,3.99)***	2.06 (1.32,3.21)**	2.54 (1.98,3.25)***
**Size of child at birth**				
Average	**Reference**	**Reference**	**Reference**	**Reference**
< average	1.66 (1.25,2.20)***	1.90 (1.48,2.45)***	1.86 (1.38,2.50)***	1.78 (1.52,2.09)***
> average	0.96 (0.74,1.23)	1.09 (0.84,1.40)	1.07 (0.79,1.46)	1.04 (0.89,1.21)
**Child sex**				
Male	1.26 (1.02,1.56)*	1.24 (1.01,1.51)*	1.35 (1.05,1.73)*	1.28 (1.23,1.46)***
Female	**Reference**	**Reference**	**Reference**	**Reference**
**Birth order**				
First	1.30 (0.80,2.11)	1.45 (0.93,2.25)	0.79 (0.39,1.62)	1.17 (0.84,1.64)
Second−third	1.31 (0.91,1.88)	1.25 (0.92,1.69)	1.01 (0.64,1.59)	1.17 (0.94,1.45)
Fourth & above	**Reference**	**Reference**	**Reference**	**Reference**
**Regional factors**				
**Regions**				
Central	**Reference**	**Reference**	**Reference**	**Reference**
Copperbelt	0.79 (0.53,1.18)	1.40 (0.86,2.28)	0.71 (0.39,1.27)	0.99 (0.73,1.33)
Eastern	1.56 (1.04,2.32)	1.88 (1.21,2.92)**	1.05 (0.63,1.76)	1.52 (1.16,1.98)**
Luapula	1.11 (0.70,1.76)	1.52 (0.91,2.55)	1.91 (1.20,3.05)**	1.50 (1.13,1.99)**
Lusaka	1.20 (0.78,1.86)	1.27 (0.78,2.06)	1.25 (0.72,2.15)	1.24 (0.93,1.65)
Northern	1.40 (0.96,2.05)	1.11 (0.67,1.82)	1.39 (0.81,2.38)	1.12 (0.83,1.50)
North-western	0.88 (0.58,1.32)	0.98 (0.60,1.60)	0.70 (0.35,1.42)	0.99 (0.73,1.33)
Southern	1.08 (0.69,1.69)	1.09 (0.69,1.71)	1.09 (0.62,1.92)	1.12 (0.83,1.50)
Western	1.24 (0.79,1.94)	0.94 (0.56,1.56)	1.05 (0.59,1.86)	0.99 (0.72,1.38)
Muchinga	–	1.27 (0.80,2.02)	1.70 (0.95,3.04)	1.43 (1.09,1.87)**

*** *P *< 0.001, ** *P* < 0.01, * *P* < 0.05, *AOR* Adjusted odds ratio, ⁋: variable not included in the multivariable analysis due to univariate *P* > 0.20

Children of adult mothers (20–34 years) had a significant lower likelihood of U5M compared to children of teenage mothers (15–19 years). Mother’s educational status and U5M has no association after adjusting for covariates. Compared to the richest households, the likelihood of U5MR was lower by 23, 27, and 19%, among the poorest, poorer, and middle households, respectively and the difference was significant only between the richest and poorer categories (AOR, 0.73, 95% CI, 0.55–0.98). Under-five mortality was 21% lower (AOR, 0.79, 95% CI, 0.67–0.93) among children in rural area than their urban counterparts, and the difference was statistically significant (Table 3).

Under-five mortality was associated with antenatal care (ANC) visit of the index child pregnancy. The likelihood of U5M was 3.17 times (AOR, 3.17, 95% CI, 2.74–3.67) higher among children of mothers who had not attended any ANC compared to those who had at least one ANC visit. No association was identified between timing in weeks (< 12 vs >  12) of the first ANC visit and U5MR in Zambia. The likelihood of U5M was higher among home-delivered children than health facility delivered under a skilled health care provider, but the difference was not significant (*P* > 0.05).

The likelihood of U5M was 2.54 times higher among multiple birth children compared to singleton birth, and the difference was statistically significant (AOR, 2.54, 95% CI, 1.98–3.25). Compared to average birth weight born children, U5M was 1.78 times (AOR, 1.78, 95% CI, 1.52–2.09) higher among smaller than average birth weight children and the difference was significant. Gender had a significant association with U5M. Male children were 1.28 times more likely to die before celebrating their fifth birthday than female counterparts (AOR, 1.28, 95% CI, 1.23–1.46). Similarly, the likelihood of U5M was 1.17 times higher among first to third birth orders than fourth and above. However, the differences were not statistically significant (*P > 0.05*).

The difference in the likelihood of U5MR among the different regions of Zambia indicated that pooled U5M was significantly higher in the Eastern (AOR, 1.52, 95% CI, 1.16–1.98), Luapula (AOR, 1.50, 95% CI, 1.13–1.99), and Muchinga (AOR, 1.43, 95% CI, 1.09–1.87) provinces compared to the Central province (Table 3).

### Trends of under-five mortality rate from 2007 to 2018 in Zambia

**Fig. 1:**
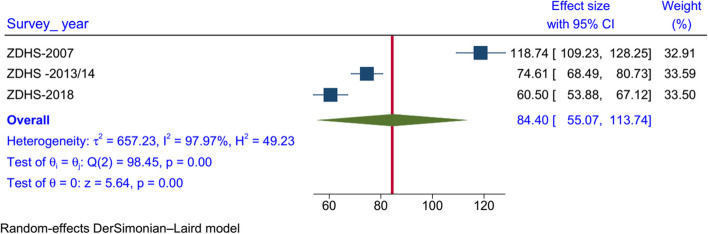
Forest plot of the national U5MR in Zambia, using data of the recent three Zambia demographic health surveys with its 95% CI

The pooled average U5MR in Zambia was 84.40 deaths per 1000 live births. The trend on the proportion of U5MR has shown a significant decline between 2007 and 2018, with a decrease of 37% between 2007 vs 2013–14 surveys, 19% between 2018 vs 2013–14 surveys, and the overall 15 years decline of U5MR was 49% between 2018 vs 2007 Zambia Demographic Health Survey (ZDHS) (Fig. 1).

**Fig. 2:**
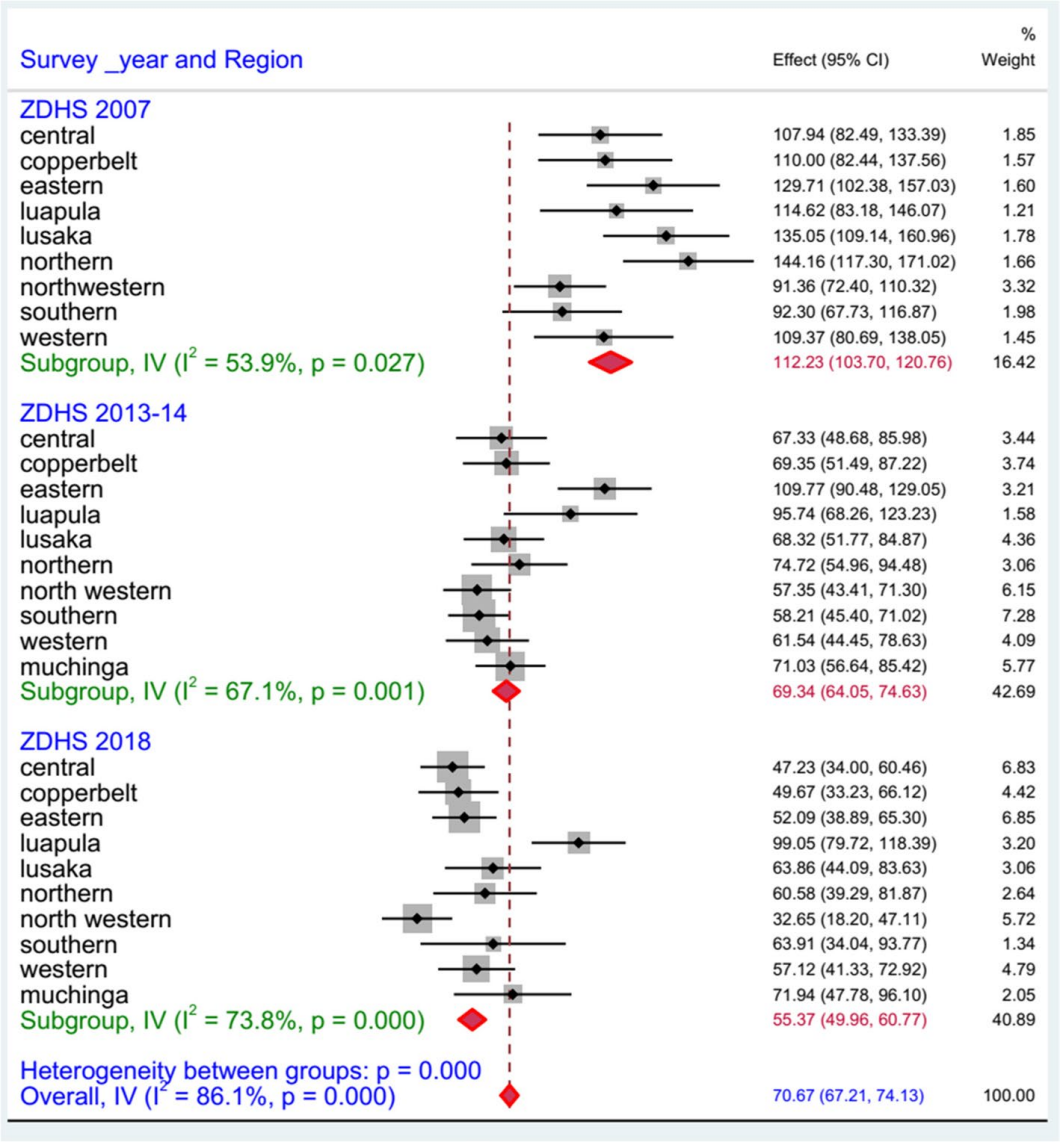
Forest plot on U5MR by provinces in Zambia using the recent ZDHS 2007, 2013–14, and 2018

Figure 2 indicates the pooled prevalence of U5MR by region in Zambia. The average U5MR across the nine regions in 2007 was 112.23 (95% CI: 103.70–120.76). A new province (Muchinga) was introduced from the Northern and Eastern districts after the 2007 survey, and the estimate of U5MR in 10 regions in the subsequent 2013–14 and 2018 survey was 69.34 (95% CI: 64.05–74.63) and 55.37 (95% CI: 49.96–60.77), respectively.

Northern, Lusaka, Luapula, and the Eastern regions were the highest proportion of U5M in the 2007 survey, and this remained high in Eastern and Luapula, and Luapula in the 2013–14 and 2018 surveys, respectively.

## Discussion

In the past years, Zambia has made a remarkable gain on child survival (49% reduction on U5MR between 2007 and 2018) in line with global success [2]. However, U5MR remains high (61/1000 live births) and the rate of decline was not uniform across the provinces in the country (Figs. 1 and 2). The highest U5MR was in the Luapula province (99.1/1000 live birth) and the lowest was in the North-Western province (32.7/1000 live births) (Fig. 2). Possible explanations for this decline could be attributed to different interventions related to malaria control [9, 12, 21], HIV control [8], and strengthening healthcare and access to key maternal and child health interventions [12, 22]. Therefore, more efforts are needed to scale up the decline and achieve the SDG3.

### Maternal characteristics and under-five mortality in Zambia

Eliminating the avoidable causes of U5M requires information on the prominent associated factors of deaths. In this study, U5M was associated with the age of mothers. This finding is consistent with studies from low and middle-income countries, which stated that children of young mothers are disadvantaged [16, 23–25], contrary to other studies [5, 26]. The likelihood of U5M was significantly lower by 26 to 39% among children born from mothers 20–34 years old compared to children of young (≤ 19 years) mothers (Table 3). This is consistent with the available literature witnessed teenage pregnancy as a risk factor for child mortality [27, 28], despite the others [26, 29]. This difference may be due to low knowledge and income among young mothers in Zambia which could bring an economic gap for lower use of maternal and child health services and young inexperienced mothers may have been encountering difficulty of proper baby caring which used to get assistance from mother-in-law, but due to the economic crisis, these practices have been getting eroded and inexperienced mothers are taking care of their children [27]. Furthermore, teenage mothers may have delayed health-seeking behavior due to shyness of early age pregnancy.

Multivariable analysis indicated that maternal education was not a significant predictor of U5M in Zambia (Table 3). This is despite the available literature that indicates maternal educational level is an important factor influencing U5M, with a better knowledge of child health conditions and favorable health-seeking behavior. Similarly, women with a higher level of education have greater decision-making autonomy concerning their children’s health and well-being [14, 15]. Therefore, further study is needed to identify the factors on U5M and mother’s knowledge in Zambia. Moreover, the likelihood of U5M was lower among lower economic status families than children from the wealthy high-income families. This is in line with the previous studies on U5M [5, 30], though a few suggest otherwise [15]. A possible explanation for this difference could be; child mortality was 80% higher among HIV patients in Zambia [8], and the prevalence of HIV may be higher in urban non-conservative societies than rural areas. Another reason could be, the DHS tools to classify household wealth based on assets including (television, radio, fridge, car, bicycle, motorcycle, electricity and others) may be another concern of using as a predictor for child health in Zambia different from the Mosley and Chen framework [19].

Unlike findings of many literatures that revealed U5M is higher among urban residents [5, 27, 31], the present study indicated the likelihood of U5M was 21% lower among rural residents than urban counterparts (Table 3). This could be due to higher HIV/AIDS and other comorbidities of U5M in the urban places [2, 8], or better access through the use of outreach services, improved awareness of the community to children health, and user fee exemptions on costs incurred towards lives of the children in the rural places of Zambia [12], similar to other countries [6, 22].

The likelihood of U5M was 3.17 times higher among children born from mothers who had not attended any ANC compared to children from mothers who had at least one ANC visit. A possible explanation for this could be that education offered during pregnancy empowered mothers on post-delivery child care and survival. Even though the likelihood of U5M was 8% higher among home delivery children, the difference was not significant. This is congruent to the findings from Chad and Mali [32].

### Child characteristics and under-five mortality in Zambia

Consistent with the findings of other reports, this study also found a strong association between multiple-birth and U5M [24, 30], albeit perhaps different from Dendup T. and colleagues report [25]. Moreover, U5M was higher among lower than average birth weight children compared to their average counterparts [27, 32]. The possible mechanism is that multiple births are at higher risk of neonatal and infant mortality [32, 33].

Gender was a significant predictor of U5M in Zambia. The likelihood of U5M was 1.28 times higher among male children than females, and the difference was significant [27, 33]. Therefore, the national level policy implication is required to include this target.

Finally, this study found that parity is a predictor of U5MR in Zambia, which is alike with the results of other studies [5, 24], but contrary to a finding by Kayode, GA [27].. U5M was 1.17 times higher among first birth and second-third births compared to fourth and above birth orders (Table 3). A possible mechanism for this could be due to lack of experience and the high risk of adverse birth outcomes like underweight are more common among young mothers, leading to higher U5M [23]. Also, this could be due to the mother’s poor nutritional status,which resulted in adverse birth outcomes.

This study also revealed that geographical region (location of residence) is a significant predictor of U5M in Zambia similar to studies from other countries [5, 25, 27, 30]. Compared to the Central province, U5M was significantly higher in the Eastern, Luapula and Muchinga (Table 3). This may be due to the greater geographical extension of these regions from the central part. This discrepancy could be because many households in different regions might have obtained different levels of children’s survival intervention programs and policies, and regions would have responded in different ways to ensure the sustainability of the intervention programs. Therefore, these regional disparities should be carefully evaluated and set as primary intervention targets to bring the national goal towards SDG3.

### Strengths and Limitations

The use of a multi-survey population-based cross-sectional study with large data sets that were selected randomly reflects the true study population, making the results generalizable. Segregated and pooled analysis by survey year, predictor variables and region are essential parameters for particular program evaluation and target policy management. Bias due to differences in survey time points and cross-sectional nature cannot be affirmed as a cause-effect relationship.

## Conclusion

Despite the decline, U5MR remains high in Zambia and thus demands improvements. The burden of U5M was higher among mothers of young age, who did not attend ANC during the index pregnancy, rich household, multiple births, smaller than average birth size, male children, and the Eastern, Luapula, and Muchinga regions of Zambia. Therefore, providing affordable and safe lifesaving interventions for children and mothers tailored to disparities at subnational health planning levels is paramount.

Furthermore this study has provided an important insights for further research on association of U5M with place of residence and household wealth in Zambia, a different finding from the existing literature which might help for a country-specific maternal health program interventions.

## Supplementary Information

**Additional file 1: Table A**: Univariate analysis of U5M and associated factors in Zambia, using the recent three ZDHS surveys.

**Additional file 2: Figure A**. Trends of U5M among the regions of Zambia over the recent three Zambia Demographic and Health Surveys.

**Additional file 3: Figure B**: Spatial distribution of under-five mortality rate per 1000 live births by regions of Zambia using data 2007, 2013-14, and 2018 Zambia Demographic and Health Surveys. Source: the map was produced by the authors using GeoDa 1.18.0.

## Data Availability

Data sets used in the analysis are publicly available and can be accessed online through application to MEASURE DHS. Analysis syntaxes and outputs generated for the study can be made available upon request to the corresponding author.

## Abbreviations

U5M: Under Five Mortality
SSA: Sub-Saharan Africa
SDG: Sustainable Development Goal
DHS: Demographic and Health Survey
ZDHS: Zambia Demographic and Health Survey
OR: Odd’s Ratio
AOR: Adjusted Odd’s Ratio
CI: Confidence Interval
HIV: Human Immunodeficiency Virus
AIDS: Acquired Immune Deficiency Syndrome
ANC: Antenatal care
