# Supplementary material for: Trends of under-five mortality and associated risk factors in Zambia: a multi survey analysis between 2007 and 2018
Source: BMC Pediatr. 2022 Jun 13;22:341. doi: 10.1186/s12887-022-03362-7 (PMC9190164; doi:10.1186/s12887-022-03362-7)
Supplement: Supplementary file 1 — Additional file 1: Table A: Univariate analysis of U5M and associated factors in Zambia, using the recent three ZDHS surveys. [file 12887_2022_3362_MOESM1_ESM.docx]

**Table A: Univariate analysis of U5M and associated factors in Zambia, using the recent three ZDHS surveys**

|  | **ZDHS-2007** | **ZDHS-2013-14** | **ZDHS-2018** | **Pooled**  **ZDHS-2007 to 2018** |
| --- | --- | --- | --- | --- |
| **Variables** | **cORs (95% CI)** | **cORs (95%CI)** | **cORs( 95%CI)** | **cORs( 95%CI)** |
| **Maternal Factors** |  |  |  |  |
| **Maternal age** |  |  |  |  |
| 15-19 | **Reference** | **Reference** | **Reference** | **Reference** |
| 20-24 | 1.06(0.72, 1.57) | 0.82(0.57,1.17) | 1.29(0.75,2.21) | 1.04(0.81,1.33) |
| 25-29 | 0.99(0.68,1.43) | 0.72(0.50,1.04) | 0.83(0.52,1.34) | 0.88(0.70,1.11) |
| 30-34 | 0.89(0.57,1.40) | 0.75(0.52,1.07) | 0.85(0.52,1.40) | 0.85(0.67,1.09) |
| 35-39 | 1.10(0.69,1.76) | 0.87(0.58,1.31) | 0.92(0.52,1.64) | 0.96(0.74,1.26) |
| 40-49 | 1.31(0.77,2.24) | 0.94(0.61,1.44) | 1.09(0.62,1.90) | 1.11(0.84,1.48) |
| **Marital status** |  |  |  |  |
| Married | **Reference** | **Reference** | **Reference** | **Reference** |
| Not married | 0.93(0.64,1.36) | 0.87(0.62,1.23) | 1.24(0.82,1.87) | 0.94(0.75,1.18) |
| **Maternal education** |  |  |  |  |
| No education | 1.12(0.80,1.57) | 1.24(0.95,1.63) | 0.96(0.67,1.38) | 1.22(1.02,1.47)* |
| Primary | 1.16(0.93,1.46) | 1.05(0.85,1.30) | 1.02(0.73,1.42) | 1.16(1.01,1.34)* |
| Secondary & higher | **Reference** | **Reference** | **Reference** | **Reference** |
| **Maternal occupation** |  |  |  |  |
| Not working | **Reference** | **Reference** | **Reference** | **Reference** |
| Working | 1.10(0.91,1.33) | 0.96(0.79,1.16) | 1.01(0.72,1.41) | 1.04(0.91,1.18) |
| **Wealth index** |  |  |  |  |
| Poorest | 0.92(0.68,1.24) | 1.26(0.90,1.78) | 1.02(0.68,1.53) | 1.08(0.88,1.32) |
| Poorer | 0.92(0.69,1.23) | 1.15(0.81,1.62) | 0.83(0.54,1.26) | 0.99(0.81,1.22) |
| Middle | 0.93(0.71,1.23) | 1.10(0.78,1.54) | 0.96(0.62,1.50) | 1.03(0.84,1.26) |
| Richer | 1.11(0.83,1.47) | 1.30(0.89,1.90) | 1.16(0.64,2.09) | 1.21(0.96,1.53) |
| Richest | **Reference** | **Reference** | **Reference** | **Reference** |
| **Place of residence** |  |  |  |  |
| Urban | **Reference** | **Reference** | **Reference** | **Reference** |
| Rural | 0.84(0.69,1.02) | 0.98(0.79,1.20) | 0.92(0.69,1.22) | 0.95(0.83,1.08) |
| **ANC visits** |  |  |  |  |
| Had at least one ANC visits | **Reference** | Reference | **Reference** | **Reference** |
| No ANC | 3.11(2.64,3.66)*** | 3.77(3.15,4.51)*** | 3.72(2.86,4.85)*** | 3.69(3.30,4.13)*** |
| **Timing of the first ANC visit** |  |  |  |  |
| < 12 weeks | **Reference** | Reference | **Reference** | **Reference** |
| > 12 weeks | 2.07(1.47,2.91)*** | 1.82(1.38,2.39)*** | 1.57(1.18,2.08)** | 1.93(1.63,2.28)*** |
| **Place of delivery** |  |  |  |  |
| Institutional delivery | **Reference** | Reference | **Reference** | **Reference** |
| Home delivery | 0.95(0.77,1.67) | 1.15(0.94,1.40) | 1.13(0.85,1.49) | 1.23(1.09,1.39)** |
| **Child Characteristics** |  |  |  |  |
| **Birth type** |  |  |  |  |
| Singleton | **Reference** | **Reference** | **Reference** | **Reference** |
| Multiple birth | 3.68(2.53,5.35)*** | 4.33(3.02,6.19)*** | 4.15(2.64,6.51)*** | 4.21(3.37,5.26)*** |
| **Size of child at birth** |  |  |  |  |
| Average | **Reference** | Reference | **Reference** | **Reference** |
| < average | 1.92(1.46,2.53)*** | 2.13(1.64,2.75)*** | 2.01(1.47,2.74)*** | 2.04(1.74,2.40)*** |
| > average | 0.93(0.74,1.1.18) | 1.02(0.79)2.75 | 1.09(0.80,1.48) | 1.04(0.89,1.21) |
| **Child sex** |  |  |  |  |
| Male | 1.27(1.04,1.54)** | 1.24(1.03,1.49)* | 1.33(1.01,1.73)* | 1.26(1.12,1.43)*** |
| Female | **Reference** | **Reference** | **Reference** | **Reference** |
| **Birth order** |  |  |  |  |
| First | 1.28(0.98,1.68) | 1.35(1.08,1.70)** | 1.25(0.93,1.68) | 1.25(1.08,1.45)** |
| Second−third | 1.11(0.88,1.40) | 0.98(0.78,1.23) | 1.12(0.87,1.44) | 1.05(0.92,1.20) |
| Fourth & above | **Reference** | **Reference** | **Reference** | **Reference** |
| **Regional factors** |  |  |  |  |
| **Regions** |  |  |  |  |
| Central | **Reference** | **Reference** | **Reference** | **Reference** |
| Copperbelt | 1.08(0.76,1.54) | 1.20(0.75,1.92) | 0.78(0.46,1.32) | 1.07(0.81,1.40) |
| Eastern | 1.31(0.93,1.84) | 1.69(1.09,2.60)* | 1.06(0.66,1.70) | 1.40(1.08,1.82)* |
| Luapula | 1.01(0.66,1.56) | 1.40(0.84,2.34) | 1.94(1.26,2.98)** | 1.38(1.05,1.82)* |
| Lusaka | 1.36(0.97,1.92) | 1.16(0.73,1.86) | 1.44(0.87,2.40) | 1.24(0.94,1.62) |
| Northern | 0.87(0.60,1.26) | 1.16(0.72,1.86) | 1.22(0.73,2.05) | 1.03(0.78,1.37) |
| North-western | 0.98(0.66,1.46) | 0.91(0.57,1.46) | 0.68(0.36,1.30) | 1.00(0.74,1.35) |
| Southern | 1.04(0.70,1.56) | 1.04(0.67,1.61) | 1.26(0.68,2.31) | 1.01(0.76,1.35) |
| Western | 1.45(1.04,2.02) | 0.89(0.54,1.45) | 1.09(0.64,1.86) | 0.78(0.57,1.08) |
| Muchinga | - | 1.15(0.73,1.79) | 1.57(0.91,2.71) | 1.61(1.22,2.12)** |

*** *P*<0.001, ** *P*<0.001, * *P*<0.05, cOR: Crud odds ratio
