# Supplementary material for: Trends of under-five mortality and associated risk factors in Zambia: a multi survey analysis between 2007 and 2018
Source: BMC Pediatr. 2022 Jun 13;22:341. doi: 10.1186/s12887-022-03362-7 (PMC9190164; doi:10.1186/s12887-022-03362-7)
Supplement: Supplementary file 2 — Additional file 2: Figure A. Trends of U5M among the regions of Zambia over the recent three Zambia Demographic and Health Surveys. [file 12887_2022_3362_MOESM2_ESM.docx]

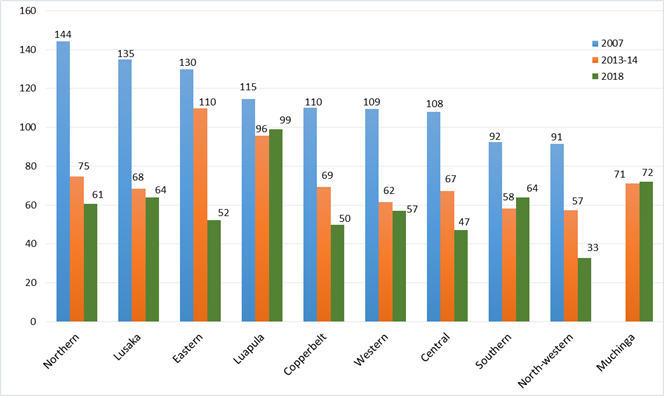


Figure A. Trends of U5M among the regions of Zambia over the recent three Zambia Demographic and Health Surveys.
