# Supplementary material for: Trends of under-five mortality and associated risk factors in Zambia: a multi survey analysis between 2007 and 2018
Source: BMC Pediatr. 2022 Jun 13;22:341. doi: 10.1186/s12887-022-03362-7 (PMC9190164; doi:10.1186/s12887-022-03362-7)
Supplement: Supplementary file 3 — Additional file 3: Figure B: Spatial distribution of under-five mortality rate per 1000 live births by regions of Zambia using data 2007, 2013-14, and 2018 Zambia Demographic and Health Surveys. Source: the map was produced by the authors using GeoDa 1.18.0. [file 12887_2022_3362_MOESM3_ESM.docx]

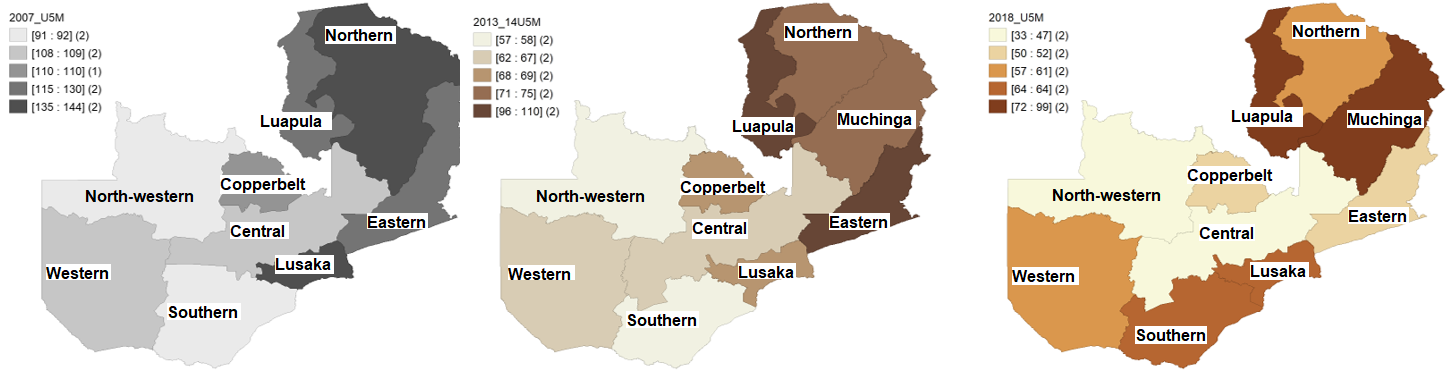


Figure B: Spatial distribution of under-five mortality rate per 1000 live births by regions of Zambia using data 2007, 2013-14, and 2018 Zambia Demographic and Health Surveys. Source: the map was produced by the authors using GeoDa 1.18.0
